# Looking up and fitting in: Team leaders' and members' behaviors and attitudes toward the environment in an MNC

**DOI:** 10.1002/hrm.22163

**Published:** 2023-01-18

**Authors:** Paul Baldassari, Sophie Eberhard, Yuan Jiang, Michael Muller‐Camen, Lisa Obereder, Michael Schiffinger, Raik Thiele

**Affiliations:** ^1^ Department of Management WU Vienna Vienna Austria; ^2^ Department of Organizational Behavior and Human Resource Management China Europe International Business School Shanghai China

**Keywords:** green HRM, green human resource management, knowledge, skills, abilities, and other characteristics, leader‐member exchange, person‐group fit, person‐supervisor fit, organizational citizenship behavior for the environment, workgroup green advocacy

## Abstract

As an emerging topic in human resource management (HRM) research, organizational citizenship behavior for the environment (OCBE) and workgroup green advocacy (WGGA) have been studied as a proxy of the environmental performance of organizations as well as a potential way for companies to assess the impact of their environmental strategies and initiatives. Viewing OCBE and WGGA as green‐focused knowledge, skills, abilities, and other characteristics and building on leader‐member exchange theory, we examined the effects of leaders' OCBE and WGGA, person‐supervisor fit (PSF), and person‐group fit (PGF) as well as their potential interactions on members' OCBE and WGGA. To minimize the potential impact of different company strategies, the study was conducted in one MNC using a sample of 269 members from 64 teams. The results revealed that PSF and especially PGF were associated with members' OCBE and WGGA, but leaders' OCBE was a stronger predictor of members' OCBE and WGGA than leaders' WGGA. Contrary to our prediction, no moderating effect of PSF or PGF was found for the associations between leaders' and members' WGGA and OCBE. Together, these findings shed light on the differential trickle‐down effects of leaders' perceptions and behaviors in the context of environmental management. As for the implications for HRM practitioners, our findings suggest companies may focus on leaders' OCBE and WGGA as well as on PSF and PGF independently as the means to shaping team members' OCBE and WGGA to support environmental strategies.

## 1 INTRODUCTION

The burgeoning fields of Sustainable and Green human resource management (HRM) reflect HRM scholars' efforts in expanding from the extant perspectives and frameworks that center on HRM's contribution to business strategy and economic performance to those that account for broader, sustainability‐related goals (Aust et al., 2020; Cooke et al., 2021; Ren & Jackson, 2020). The underlying logic is that to succeed in implementing environmental strategies and achieving environmental objectives, corporations ought to design and implement Green HRM policies and practices to manage their environmental processes and performance (Jackson et al., 2011). Moreover, in recognition of the drawbacks to focusing solely on executives and overlooking rank‐and‐file employees when measuring pro‐environmental attitudes and behaviors (Aguinis & Glavas, 2012; Robertson & Carleton, 2018), recent Green HRM studies have explored the influence of Green HRM practices such as training, performance management, and employee involvement on employee green‐related behavior and other desirable outcomes (Tang et al., 2018; Usman et al., 2023).

Nevertheless, too few companies have adopted Green HRM and related measures in their formal policies and practices (Obereder et al., 2022). Moreover, even when Green HRM policies are in place, most companies still rely on employees' broad voluntary efforts to achieve their environmental sustainability goals (Andersson et al., 2013; Ones & Dilchert, 2012). Further, from an HRM perspective, Green HRM interventions and human resource development efforts in most companies can be directed realistically at only a limited number of employees such as leaders of work teams. The key is whether the results of such greening initiatives by HRM trickle down to these leaders' subordinates. For instance, in the domain of environmental training (Renwick et al., 2013), a related question is how team leaders, once trained, can maximize the return on training by coaching their teams.

In fact, examining such trickle‐down effects from higher‐level leaders to lower‐level employees would also fill a gap in the literature on pro‐environmental behavior. In particular, a trickle‐down effect typically implies multilevel dynamics, a topic that has received less attention in past research (Norton et al., 2015). As a result, few studies have adopted a multilevel perspective and considered higher‐level conditions as antecedents of employees' green behavior (see recent exceptions, Jiang et al., 2022). With a better understanding of employee green behavior in a multilevel framework, such behavior is likelier to be promoted via various managerial behaviors and HR practices (Dumont et al., 2017).

Additionally, past research has demonstrated the importance of certain leadership styles (Tu et al., 2023) and behavior in promoting corresponding green behavior by members, especially when leaders and members frequently interact (Yaffe & Kark, 2011). Nevertheless, almost no study has closely examined whether and how employees' perceived fit with their social contexts affects how they react to green behavior from their leaders. Yet, several fit indices, for instance, represent central HRM variables (Werbel & DeMarie, 2005) and may strengthen the positive influence of HRM interventions on eco‐friendly behaviors (Zhao et al., 2021). Surprisingly, to our knowledge, no research has integrated the perceived fit of members with both their leaders and their group when investigating how such perceptions of “fit” potentially affect the consonance between leaders' and members' environmental behavior.

Consequently, our study raises two main research questions. First, how do a leader's eco‐friendly behaviors and attitudes trickle down to members within a work team context? Second, how is this potential dissemination of pro‐environmental behaviors and attitudes from team leader to members moderated by members' perceived fit with the team or with the leader?

Recognizing the importance of employees' roles in a company's environmental performance and the voluntary nature of eco‐friendly behavior, we investigated employees' voluntary pro‐environmental behavior, which is also termed organizational citizenship behavior for the environment (OCBE) (Boiral, 2009; Boiral & Paillé, 2012). Further, because employees' behavior is also strongly shaped by their perceptions of the immediate work environment, that is, one's work group or team, we examined their perceptions of work group green advocacy (WGGA) (Kim et al., 2017) as a facet of psychological climate that targets environmental issues and promotes pro‐environmental behavior. Like OCBE (Paillé et al., 2014), WGGA is amenable to Green HRM practices (Sabokro et al., 2021) and represents a relevant concept for an organization's overall greening process (Dumont et al., 2017). However, these concepts touch on different aspects insofar as OCBE focuses on behavior while WGGA is more of an attitudinal construct. More importantly, from a human capital resource perspective, individuals' knowledge, skills, abilities, and other characteristics (KSAOs) aggregate to create the human capital resource in an organization, an aggregation that helps it achieve its ultimate performance goals (Ployhart & Moliterno, 2011). Hence, we view employees' positive attitudes toward and voluntary effort in green initiatives and behavior as a manifestation of individual KSAOs in the domain of environmental sustainability that collectively transforms into firm‐level environmental performance.

We turn next to the context of work teams because they are employees' immediate work environment, and organizations have extensively adopted team‐based structures to perform and coordinate work (Mathieu, Gallagher, et al., 2019). Because they hold more power and resources, team leaders generally serve as role models to guide team members' behavior. In addition, their positive assessment of the team's pro‐environmental orientation casts normative cues regarding desirable attitudes and behaviors. Emotional and instrumental support shown by supervisors has been identified as important antecedents of eco‐friendly behaviors in their subordinates (Paillé et al., 2020). In acknowledging the substantially influential role of team leaders, we hence undertook to explore a multilevel process through which their OCBE and WGGA influence individual employees' OCBE and WGGA. In doing so, we address a gap in the literature by examining multilevel relationships around pro‐environmental behavior (e.g., Norton et al., 2015). In particular, we adopted the leader‐member exchange (LMX) theory as our overarching framework, with social exchange as the underlying mechanism for the relationships of attitudes and behaviors between team leaders and members.

We further suggest that the extent to which team leaders' pro‐environmental behaviors and attitudes are accepted by their members may be partially determined by their perceived (mis)fit with their leaders and team members as a whole in light of the congruence of values and norms in the environment (Jansen & Kristof‐Brown, 2006). To account for this, we wanted to extend existing research by incorporating the potential positive effects of the perceived fit between team members and their leaders. More specifically, we used a sample of employees from an MNC operating in multiple countries to test the moderating effect of person‐supervisor fit (PSF) and person‐group fit (PGF) on the positive relationships between leaders' and members' OCBE and WGGA in teams. The firm had established a formal set of environmental strategies and practices, with considerable discretion left to managers and employees in performing voluntary green behavior. This context makes a study of voluntary green behaviors suitable and relevant because we were able to test our hypothesis by using data from different countries while benefiting from a consistent organizational environment (Andersson et al., 2005).

We make two contributions to the literature on Green HRM and green behavior in organizations. First, in recognizing the prevalence of team‐based structures in organizations and the power of such structures in molding employees' attitudes and behaviors (Mathieu, Gallagher, et al., 2019), we focused on pro‐environmental behaviors and attitudes as the manifestation of individual KSAOs from a human capital resource perspective in the context of environmental management. Responding to a recent call for more multilevel research on pro‐environmental behavior (Norton et al., 2015), our simultaneous examination of the trickle‐down effects of these behaviors and attitudes advances our understanding of how a multilevel process can potentially foster the development of green KSAOs in teams.

**FIGURE 1:**
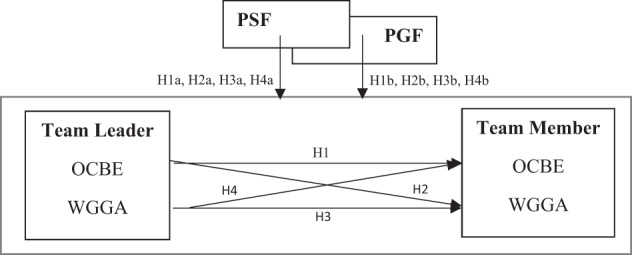
Hypothesized model

Second, we investigated whether the proposed trickle‐down effects vary across members when they perceive different levels of fit with their supervisors and teams. Doing so extends the fit research to the context of environmental sustainability in which organizational members serve as good citizens to safeguard the environment for society at large. The fit literature has been well‐linked to organizational citizenship behavior in organizations (Kristof‐Brown et al., 2018). However, to our knowledge, our study is the first to empirically examine the effects of fit variables on individuals' green‐focused perception and behavior in a team context, highlighting them as potentially central target variables for (Green) HRM research and practice. Our theoretical model is depicted in Figure 1.

## 2 THEORETICAL BACKGROUND

### 2.1 Green behavior at work

Green behavior at work can be defined as behavior in the work context that contributes to environmental sustainability or hinders it (Ones & Dilchert, 2012). An essential distinction in green behaviors at work can be made between required and voluntary behavior (Norton et al., 2015). First, required green behavior is performed as part of the employee's job duties and comprises “adhering to organizational policies, changing methods of work including choosing responsible alternatives, and creating sustainable products and processes” (Norton et al., 2015, p. 105). Second, in case employees show an environmental commitment that exceeds organizational requirements, green behavior can be considered as a voluntary type of behavior. Research has typically chosen this second type of behavior when examining green behavior in the workplace, treating voluntary green behavior as an extension of organizational citizenship behavior (Norton et al., 2015; Yuriev et al., 2018).

Boiral (2009) as well as Daily et al. (2009) were among the first to propose that OCBE can help an organization achieve its environmental objectives. This perspective drawing on discretionary initiatives at the individual level challenged the common research focus on the formal, managerial aspects of environmental management (Boiral, 2009). OCBE refers to voluntary behavior that is not formally rewarded or recognized, thus it can be considered extra‐role behavior (Boiral, 2009). Employees can show various types of OCBE such as “…sharing knowledge to prevent pollution in the workplace, suggesting solutions aimed at reducing waste, … and collaborating with the environmental department to implement green technology.” (Boiral & Paillé, 2012, pp. 431–432).

In contrast, Kim et al. (2017) defined WGGA “…as the extent to which work group members openly discuss environmental sustainability, share relevant knowledge, and communicate their various views in order to encourage others to engage in eco‐friendly behavior.” (p. 1342). WGGA can thus be regarded as a type of psychological climate (James et al., 2008) relating to green activism (Briscoe & Gupta, 2016); it proactively communicates peers' expectations about the importance of proactive green behavior and represents an environmental commitment that leads to desired outcomes (Kim et al., 2017). Individual‐level WGGA refers to one's perception of such a pro‐environment climate in his or her team. In this study, both OCBE and WGGA are conceptualized as individual‐level constructs. Given that team leaders play a critical part in their team members' immediate social environments and thus influence members' perceptions and behaviors through social interactions and exchanges (Bandura, 1999), we drew on LMX theory to build our overarching framework and lay the ground for hypotheses development.

### 2.2 Leader‐member exchange theory

Essentially, LMX theory represents a relational approach to understanding the leadership process (Graen & Uhl‐Bien, 1995; Liden et al., 2016). Originally coined as “Vertical Dyad Linkage Model” (Dansereau et al., 1975; Graen & Cashman, 1975; Liden & Graen, 1980), the basic tenet of LMX is that leaders develop dyadic relationships with their followers. Based on these relationships, leadership processes unfold and engender the subsequent attitudes and behaviors of both leaders and followers. As one of the most prominent approaches to leadership, previous meta‐analyses reported fairly consistent results regarding the positive impacts of LMX on an array of attitudinal and behavioral outcomes (Dulebohn et al., 2012; Gerstner & Day, 1997; Ilies et al., 2007).

At the core of LMX is the social exchange process between a leader and a follower (Erdogan & Liden, 2002; Liden & Sparrowe, 1997). Social exchange theory differentiates between economic and social exchanges (Blau, 1964), with the former focusing on material transactions and the latter on enduring mutual obligations and commitment. A primary guideline for social exchange is the norm of reciprocity (Gouldner, 1960). As summarized by Cropanzano and Mitchell (2005), reciprocity can stem from interdependent exchanges and/or general cultural expectations and mandates. By engaging in reciprocal exchanges with their leaders, members develop trust in and beneficial relationships with the leaders.

Another aspect of LMX is the differentiation of the relationships developed between a leader and his or her subordinates. Earlier work stressed the in‐group versus out‐group distinction among these relationships and associated differences in resource allocation and subordinate attitudes and behaviors (Dansereau et al., 1975; Graen & Cashman, 1975; Liden & Graen, 1980). Later, scholars refined the model by treating exchange relationships as a continuum (ranging from low to high) rather than as demarcating a clear boundary between the in‐group and the out‐group (Henderson et al., 2009; Yu et al., 2018).

Building on the LMX framework, we argue that team leaders' OCBE and WGGA engage team members in a social exchange process, which in turn elicits their own OCBE and WGGA. To unpack the relationships of attitudes and behaviors between team leaders and their members, we relied on the supervisory support highlighted in both LMX (Graen & Uhl‐Bien, 1995) and social exchange theory (Bandura, 1999; Blau, 1964). Specifically, we considered leaders' OCBE as one type of instrumental support and their WGGA as one type of emotional support, both of which create a social exchange context for members (Paillé et al., 2020). More precisely, witnessing a leader's OCBE represents instrumental support by serving as a type of task assistance or instruction as well as delivering task‐related knowledge (Mathieu, Eschleman, & Cheng, 2019) in a way that makes how a superior does things appear as the best practice or benchmark for imitation. In parallel, the leader's positive assessment of open communication and advocacy of environmental concerns within the team (WGGA) can provide socioemotional resources in the form of anticipated or perceived understanding and esteem for sharing these concerns and behaving in accordance with the underlying (implicit) values, thereby providing emotional support (Mathieu, Eschleman, & Cheng, 2019; Mathieu, Gallagher, et al., 2019). Because the success of workplace greening hinges on the joint efforts of both team leaders and members, this creates a basis for the reciprocal interdependence and transactions featured in the social exchange process.

Adopting an overarching framework of LMX rooted in social exchange theory, we next explain how team leaders' OCBE and WGGA are associated with team members' OCBE and WGGA. Because all members in a team observe a leader's OCBE and WGGA, we focused our theorizing on their influence on individual members instead of on different subgroups within the team (i.e., in‐group and out‐group).

### 2.3 The impacts of leader OCBE and WGGA

A major antecedent of OCBE is the work environment and especially the social context (Norton et al., 2015). In a work‐team setting, the team leader serves as the primary representative of the organization; as such, the team leader may have considerable influence on members' OCBE. One powerful way to shape members' behavior is through the leader's own display of the desirable behavior expected of members (e.g., A. Kim et al., 2017; Robertson & Barling, 2013). From a social exchange perspective, observing the leader's effort to create a more eco‐friendly work environment will likely inspire members to reciprocate the same behavior because it is a joint effort and that will benefit everyone in the organization. Further, from an LMX perspective, leaders' OCBE operates as critical resources for members by providing instrumental support in the form of the relevant knowledge and behavioral modeling needed for them to learn and demonstrate the same behavior in return (Shao et al., 2022).

In the environmental management context, supervisory support perceived by employees underpins the social exchange process, which ultimately facilitates employees' eco‐initiatives and pro‐environmental behaviors (Paillé et al., 2020). Combining with previous studies reporting a positive association between supervisors' and employees' pro‐environmental behaviors (e.g., A. Kim et al., 2017; Robertson & Barling, 2013), we propose:

*Team leaders' OCBE is positively associated with team members' OCBE*.

In line with this reasoning, we contend that in addition to serving as members' behavioral models, team leaders can also leverage their OCBE to build positive evaluations and attitudes toward environmental sustainability in their work environments. In the course of perceiving their leaders' OCBE as instrumental support, team members are inclined to reciprocate by advocating for the perceived behaviors as a way to show their shared concern and to develop high‐quality relationships with the leader (A. Kim et al., 2017). Parallel to the linkage between perceived support and attitudinal outcomes favorable to the organization (Rhoades & Eisenberger, 2002; Rockstuhl et al., 2020), leaders' OCBE (as instrumental support) is likely associated with members' WGGA (as a favorable attitude to the organization). Therefore, we propose:

*Team leaders' OCBE is positively associated with team members' WGGA*.

Similar to leaders' actions, leaders' expressing a supportive attitude and encouragement of environmental actions instill commitment and support in team members (Raineri & Paillé, 2016). In particular, team leaders' positive attitudes and assessments pertaining to a pro‐environmental work climate facilitate the building of such a climate. Perceiving these positive attitudes and commitment as emotional support (Paillé et al., 2020), team members are likely to be engaged in a social exchange process by also showing positive attitudes and assessments, thereby co‐creating a pro‐environmental work atmosphere.

Further, this effect could be especially important in multilevel hierarchies because supportive attitudes from top management promote mid‐level leader support and correspond with lower‐level employees' positive attitudes, enabling a multiplier effect (Park et al., 2014). We intend to address the additional research necessary to confirm the robustness of this correlation across multiple settings. Therefore, we propose:

*Team leaders' WGGA is positively associated with team members' WGGA*.

Likewise, leaders' supportive attitudes toward environmental sustainability will be viewed as emotional support by team members, thus likely eliciting their OCBE in return for their leaders' support. Supervisors' attitudes and support encourage employees' OCBE through multiple channels for exchanges. Line managers encouraging and valuing environmental protection have been shown to motivate employees (Raineri & Paillé, 2016). Graves et al. (2013) revealed a correlation in Chinese companies between environmental transformational leadership and environmental behavior. We will test the influence of team leaders' advocacy on team‐level behavior in our international setting. Therefore, we propose:

*Team leaders' WGGA is positively associated with team members' OCBE*.

### 2.4 The role of person‐supervisor and person‐group fit

PSF and PGF are two dimensions of the person‐environment fit (PEF), which refers to the compatibility between individuals and specific aspects of their work environment (Jansen & Kristof‐Brown, 2006). PEF has guided the development and implementation of HRM practices and procedures, facilitated the establishment of competencies, and safeguarded desired employee attributes and behaviors (Werbel & DeMarie, 2005). Besides this general relevance, its importance has been shown for eco‐friendly behavior, too (Zhao et al., 2021). In our study, PGF and PSF refer to value congruence as a dimension of supplementary fit, which is generally expected to interact with or modify individual behaviors and attitudes as a result of enhanced trust and attraction toward people sharing matching or similar attributes (Cable & Edwards, 2004). We propose that in the team context, perceived fit with one's own team and supervisor will play a role in strengthening or weakening the trickle‐down effects of team leaders' environment‐relevant attitudes and behaviors.

As for PSF, team leaders are not automatically perceived as role models because of their mere organizational status. This occurs only if their subordinates regard them as worthy examples who—beyond fulfilling role expectations—represent what people aspire to become and achieve (Yaffe & Kark, 2011). We propose that high PSF comes with a perception of similarity in value congruence that in turn increases the probability of a member being sympathetic to the leader (Liden et al., 2016). Such perceived similarity and enhanced liking or positive affect toward a leader positively influence LMX processes and their quality (Dulebohn et al., 2012). Consequently, we expect that, based on enhanced LMX quality, the fit between team leaders' and team members' values and norms reinforces the connection of the leader and members in terms of their perceived green climate as well as their actual green behavior.

Because of closer and more meaningful interactions that accompany such high LMX quality (Maslyn & Uhl‐Bien, 2001), we, therefore, conclude that members will be more receptive and responsive toward environmental engagement such as OCBE displayed by their leader and reciprocate even more naturally by showing the witnessed behavior themselves. Thus we posit:

*The positive association between team leaders' OCBE and team members' OCBE is amplified by higher PSF*.

Because of such felt proximity, along with the additional concomitant increase in the salience of leaders' behavior, we also expect leaders' OCBE to outshine, and seemingly signal, their entire teams' relation to the environment, which in turn advances attitudes and estimates regarding the overall psychological green climate within the team. This leads us to the following assumption:

*The positive association between team leaders OCBE and team members WGGA is amplified by higher PSF*.

Moreover, the leaders' attitudes and assessments themselves are also more directly and distinctly accessible from a close distance, which is why we expect a higher likelihood for members with strong PSF to reciprocate and adopt from their leaders a similar evaluation of and identification with the team's attitude toward the environment. Therefore, we suggest:

*The positive association between team leaders' WGGA and team members' WGGA is amplified by higher PSF*.

Lastly, when PSF is strong, members should be even more responsive toward related cues and expectations regarding their own green engagement. Such cues and expectations are often indicated by leaders' WGGA. In an effort to maintain or foster a positive connection with their supervisor (Maslyn & Uhl‐Bien, 2001), we suspect members will try to meet expectations by adopting desirable behaviors implicit to leaders' WGGA. Accordingly, we propose:

*The positive association between team leaders' WGGA and team members' OCBE is amplified by higher PSF*.

Additionally, we propose that PGF works in a similar way via value congruence and LMX quality. Even though value congruence concerns the other team members rather than the team leaders, in this case, PGF is still potentially conducive to the translation of environmental engagement, considering that it not only enhances individual satisfaction with coworkers and supervisors but also fosters group cohesion (Kristof‐Brown et al., 2005). More precisely, on the assumption that members of cohesive teams tend to also develop more profound relationships with their leader (Cogliser & Schriesheim, 2000), we expect PGF to have a positive association with LMX quality. Accordingly, PGF should equally strengthen the link between team leaders' and team members' green engagement.

By applying arguments identical to those leading to our four hypotheses on PSF, we, therefore, propose that PGF synonymously reinforces the connection of leaders' and members' OCBE and WGGA by means of a more substantial LMX quality. Therefore, we propose the following four hypotheses linked to PGF:

*The positive association between team leaders' OCBE and team members' OCBE is amplified by higher PGF*.

*The positive association between team leaders' OCBE and team members' WGGA is amplified by higher PGF*.

*The positive association between team leaders' WGGA and team members' WGGA is amplified by higher PGF*.

*The positive association between team leaders' WGGA and team members' OCBE is amplified by higher PGF*.

## 3 METHODOLOGY

### 3.1 Data collection

The study has been conducted in eight subsidiaries of a U.S. MNC in Austria, China, India, and the U.S. The company is one of the largest providers of electronics manufacturing services in the world and has established and published its environmental strategy and performance for more than 20 years. We were able to access environmental organization specifics, communication, and training materials during our study. This material supported our assumption that the MNC's leaders and team members had a general understanding of the company's environmental strategy and goals. The selection of teams was done through collaboration between representatives of the subsidiaries and the researchers. In the selection process, we targeted a range of functional, operational, and business groups within the subsidiaries to capture survey data from multiple employee cohorts. To be considered for participation, teams had to consist of at least one team member and a supervisor and had to have worked together for at least 1 year. In two subsidiaries, representatives invited participants from all eligible or randomly selected teams. In the other subsidiaries, representatives chose to select participating teams based on their workloads and availability. Our contacts within the company sent out survey requests as well as reminders via company email. All participants were guaranteed anonymity in their responses.

Our company contacts were also responsible for the allocation of team codes, which enabled the grouping of participants to their respective teams in an aggregated and anonymous form. All participants were instructed to complete the online survey during their working hours and, if possible, at the workplace. Most surveys were conducted in English. However, for Austria and China, researchers used established translation procedures (Brislin, 1990) to translate surveys into German and simplified Chinese. They were applied as default languages in the respective countries with the option to switch to English at any time.

### 3.2 Sample

The final sample consisted of 269 team members who belonged to 64 teams with one leader (and at least two team members) per team. For the hierarchical linear models, the survey responses were split into team leaders' WGGA and OCBE (Level‐2 predictor variable) and team members' WGGA and OCBE (Level‐1 criterion) as separate variables, resulting in a sample size of 269 team members. The proportion of women was 22% among team members and 18.5% among team leaders. The mean age of team members was 37.8 years ± 9.5; the mean team leaders' age was 42.8 years ± 6.3. In terms of location, 11% of the teams were based in Austria, 19% in the U.S., 31% in India, and 39% in China; the distribution of team members was as follows: 7% from Austria, 22% from the U.S., 38% from India, and 34% from China.

### 3.3 Measures

#### 3.3.1 OCBE

To assess all participants' discretionary green workplace behaviors, they completed the 10‐item OCBE scale developed by Boiral and Paillé (2012). Sample items are “I stay informed of my company's environmental initiatives” and “I voluntarily carry out environmental actions and initiatives in my daily work activities.”

#### 3.3.2 WGGA

Participants reported their perceptions of their team members' green advocacy behaviors using the WGGA scale by Kim et al. (2017). The following three items were used: (1) “Members in my work group try to convince my group members to reduce, reuse, and recycle office supplies in the workplace,” (2) “Members in my work group work with each other to create a more environmentally friendly workplace,” and (3) “Members in my work group share knowledge, information, and suggestions on workplace pollution prevention with other group members.”

#### 3.3.3 PSF

Team members' PSF was measured with the instrument developed by T. Y. Kim and Kim (2013). The three items of this scale are: (1) “The things that I value in life are similar to the things my supervisor values,” (2) “My personal values match my supervisor's values,” and (3) “My supervisor's values provide a good fit with the things that I value in life.”

#### 3.3.4 PGF

Likewise, surveys included a scale on PGF consisting of the following three items: (1) “The things I value in life are similar to the things my coworkers value,” (2) “My personal values match my coworkers' values,” and (3) “My coworkers' values provide a good fit with the things that I value in life.” The scale is based on Cable and DeRue (2002) and Greguras and Diefendorff (2009).

#### 3.3.5 Control variables

Although gender has not been consistently shown to be associated with green behavior (e.g., Ren, Tang, & Kim, 2022), women tend to express more positive attitudes toward it than men do (e.g., World Bank, 2009), so we included it on both the leader and member levels. The same observation of higher environmental awareness could be made with increasing age and education level (Gifford & Sussman, 2012; World Bank, 2009), which is why we used these as control variables, too. Finally, we included the number of team members because the size of groups has been shown to be related to their leaders' behavior and influence (Bass & Stogdill, 1990).

### 3.4 Analysis

In the first step, we examined the measurement properties of the four constructs (WGGA, OCBE, PSF, and PGF) with confirmatory factor analysis (CFA) (robust maximum likelihood estimation because all items are scored on a rating scale with at least five options; Rhemtulla et al., 2012). Regarding potential common method bias, we complemented the CFA analysis with a marker variable approach as described in Williams et al. (2010). The marker we chose was horizontal individualism, which was measured in the same way as the focal variables (i.e., with Likert scales), but is theoretically unrelated to them, making it a suitable marker variable (Simmering et al., 2015).

Testing the hypotheses involved examining the relationship between the same variables (WGGA and OCBE) for different groups (team leaders and members), so we chose factor score regression (Devlieger & Rosseel, 2017), with correlation‐preserving Ten Berge factor scores (Logan et al., 2019). The leader factor scores for WGGA and OCBE were set as Level‐2 predictors per team, the member scores for PSF and PGF are Level‐1 predictors (and the member scores for WGGA and OCBE were the dependent variables).

To test our assumptions, we ran hierarchical linear models (restricted maximum likelihood estimation) with a random intercept for the team and the previously mentioned predictors and control variables. Because our moderation hypotheses posit a cross‐level interaction (leaders' WGGA and OCBE as a Level‐2 variable and members' PSF and PGF as a Level‐1 variable), we group mean‐centered PSF and PGF, in accordance with the recommendation by Aguinis et al. (2013). In addition to this within‐team variation, we included the between‐team (Level 2) variation of PSF and PGF into the respective models, too (i.e., the team‐level deviation of PSF and PGF from the overall mean).

Because the factor scores derived from the CFA lack any intuitively interpretable metric, we standardized the four psychometric variables (WGGA, OCBE, PSF, and PGF), making those coefficients interpretable like standardized beta coefficients in ordinary least squares regression in terms of the underlying effect size. Even before standardization, the original mean of these scores was close to zero (−0.05–0.02), and their standard deviation was close to one (0.99–1.05). Members' age and number of team members were grand‐mean‐centered (and not standardized).

The software used included IBM SPSS v22 (for initial data exploration and descriptive analyses) and R version 4.2.0 for the measurement model and hypothesis testing analyses, with the packages lavaan (v06‐11), semTools (v0.5–6), careless (v1.2.1), psych (v2.2.9), lme4 (v1.1–29), lmerTest (v3.1–3), misty (v0.4.6), performance (v0.9.0), and REndo (v2.4.6).

### 3.5 Results

An initial CFA model (with 333 responses from 269 team members and 64 team leaders) resulted in somewhat partly unsatisfactory fit indices (*χ* ^2^_[146]_ = 472.6, Comparative Fit Index (CFI) = 0.92, Non Normed Fit Index (NNFI) = 0.91, Root Mean Square Error of Approximation (RMSEA) = 0.091, Standardized Root Mean Square Residual (SRMR) = 0.050; robust values reported), but these improved considerably when two residual correlations for OCBE were allowed: *χ* ^2^_(144)_ = 362.7, CFI = 0.94, NNFI = 0.93, RMSEA = 0.074, SRMR = 0.046. Although the practice of allowing correlated residuals is generally not recommended, we contend it is justifiable here because it is limited to similarly worded items from the same scale,^1^ which can result in correlated residuals (Bandalos, 2021). Scale consistency was satisfactory with omega values of 0.86 for WGGA, 0.94 for OCBE, 0.93 for PSF, and 0.85 for PGF. Average variance extracted is >0.5 (WGGA: 0.69, OCBE: 0.63, PSF: 0.83, PGF: 0.67), with 0.6 as the highest squared correlation between latent variables, thus corroborating convergent and discriminatory validity.

As for common method bias, we found no evidence of method variance associated with the marker variable, based on the non‐significant difference between a model in which the method factor loads (equally) on the substantive constructs and a model in which this is not the case (Δ*χ* ^2^_(1)_ = 0.28, *p* = 0.60). Allowing unequal method factor loadings on the substantive constructs did result in improved model fit (Δ*χ* ^2^_[18]_ = 45.4, *p* < 0.01). However, there was no evidence of a biasing effect on the correlations between the substantive constructs when we compared a model with latent variable correlations fixed at the values obtained without the marker variable to a model that included the marker and freely estimated correlations (Δ*χ* ^2^_[6]_ = 0.01, *p* > 0.99). Overall, these results suggest common method bias is not a potential threat in this study.

Examining measurement invariance between team leaders and team members yielded satisfactory results, both for metric (Δ*χ* ^2^_[15]_ = 21.71, *p* = 0.12) and scalar invariance (Δ*χ* ^2^_[15]_ = 12.79, *p* = 0.62). By contrast, measurement invariance with regard to the country could not be established, but the distinction between countries is not a focal one for this study.

As for hierarchical linear modeling (HLM) results, the empty model with just a random team intercept resulted in an intraclass correlation coefficient of 0.16 for WGGA and 0.31 for OCBE. The following tables present the results of the HLM models with the additional fixed effects. We examined random slope models, too; however, this rarely resulted in better model fit but quite frequently yielded singularity issues without any change in the substantial results. All results are based on the listwise n of 269 members from 64 teams (and thus team leaders). The left part of the tables refers to the models with PSF as a moderator variable, and the right to those with PGF.

Table 1 shows the results for the first hypothesis on the association between team leaders' OCBE and team members' OCBE, with an assumed reinforcing effect of PSF (H1a) and PGF (H1b). Although leaders' OCBE emerged as a statistically significant predictor of members' OCBE (one‐tailed *p* < 0.01 and 0.03) in accordance with our H1, no appreciable moderating effect of PSF or PGF was observed, contrary to our predictions. In contrast, both PSF and PGF show a considerable association with members' OCBE.

**TABLE 1:** Hierarchical linear modeling (HLM) results for H1a/b: Effect of team leader organizational citizenship behavior for the environment (OCBE) × Person‐supervisor fit/Person‐group fit on team members' OCBE

DV: Members' OCBE	Est. (s.e.)	95% CI	*p* (2‐tail)		Est. (s.e.)	95% CI	*p* (2‐tail)
Intercept	0.026 (0.086)	−0.136/0.189	0.762	Intercept	0.010 (0.079)	−0.139/0.158	0.901
OCBE leader	0.200 (0.077)	0.053/0.347	0.012 *	OCBE leader	0.138 (0.075)	−0.004/0.279	0.069
Member PSF (within teams)	0.257 (0.047)	0.165/0.349	<0.001 ***	Member PGF (within teams)	0.310 (0.045)	0.221/0.398	<0.001 ***
Member PSF (between teams)	0.254 (0.082)	0.098/0.410	0.003 **	Member PGF (between teams)	0.371 (0.081)	0.218/0.524	<0.001 ***
Leader female	−0.169 (0.201)	−0.552/0.212	0.404	Leader female	−0.099 (0.187)	−0.455/0.256	0.600
Member female	0.047 (0.138)	−0.222/0.312	0.734	Member female	0.060 (0.132)	−0.198/0.313	0.649
Age	−0.013 (0.007)	−0.025/0.000	0.056	Age	−0.009 (0.006)	−0.021/0.003	0.155
Educational level	−0.030 (0.076)	−0.174/0.127	0.692	Educational level	−0.037 (0.073)	−0.175/0.111	0.613
Number of team members	−0.001 (0.024)	−0.047/0.045	0.966	Number of team members	−0.007 (0.022)	−0.048/0.034	0.752
OCBE leader × PSF	0.029 (0.044)	−0.058/0.117	0.517	OCBE leader × PGF	−0.011 (0.045)	−0.099/0.077	0.801
Residual team‐level variance	0.167	Cond. *R* ^2^	0.424	Residual team‐level variance	0.128	Cond. *R* ^2^	0.458
Residual member‐level variance	0.582	Marg. *R* ^2^	0.258	Residual member‐level variance	0.546	Marg. *R* ^2^	0.331

The second hypothesis refers to the association between team leaders' OCBE and team members' WGGA, again positing a moderating effect of PSF (H2a) and PGF (H2b). The pattern of results is virtually identical to those for H1: leaders OCBE as well as the PSF and PGF are predictive of members' WGGA. However, neither PSF nor PGF moderate the effect of leaders' OCBE (see Table 2).

**TABLE 2:** Hierarchical linear modeling (HLM) results for H2a/b: Effect of team leaders' organizational citizenship behavior for the environment (OCBE) × Person‐supervisor fit/Person‐group fit on team members' workgroup green advocacy (WGGA)

DV: Members' WGGA	Est. (s.e.)	95% CI	*p* (2‐tail)		Est. (s.e.)	95% CI	*p* (2‐tail)
Intercept	0.011 (0.072)	−0.125/0.146	0.881	Intercept	−0.001 (0.066)	−0.124/0.122	0.993
OCBE leader	0.177 (0.070)	0.045/0.310	0.014 *	OCBE leader	0.123 (0.068)	−0.005/0.250	0.073
Member PSF (within teams)	0.189 (0.055)	0.081/0.296	<0.001 ***	Member PGF (within teams)	0.311 (0.052)	0.208/0.412	<0.001 ***
Member PSF (between teams)	0.159 (0.073)	0.021/0.297	0.034 *	Member PGF (between teams)	0.282 (0.071)	0.149/0.416	<0.001 ***
Leader female	−0.314 (0.182)	−0.659/0.026	0.087	Leader female	−0.257 (0.168)	−0.575/0.059	0.131
Member female	0.206 (0.151)	−0.080/0.502	0.173	Member female	0.209 (0.142)	−0.060/0.486	0.142
Age	−0.016 (0.007)	−0.029/‐0.003	0.023 *	Age	−0.011 (0.006)	−0.024/0.001	0.081
Educational level	−0.034 (0.084)	−0.191/0.135	0.687	Educational level	−0.049 (0.079)	−0.197/0.108	0.533
Number of team members	−0.001 (0.019)	−0.037/0.036	0.973	Number of team members	−0.008 (0.017)	−0.040/0.024	0.650
OCBE leader × PSF	0.037 (0.053)	−0.065/0.140	0.480	OCBE leader × PGF	−0.021 (0.052)	−0.123/0.081	0.684
Residual team‐level variance	0.039	Cond. *R* ^2^	0.219	Residual team‐level variance	0.019	Cond. *R* ^2^	0.290
Residual member‐level variance	0.800	Marg. *R* ^2^	0.181	Residual member‐level variance	0.726	Marg. *R* ^2^	0.272

The third hypothesis addresses the effect of leaders' WGGA on members' WGGA and its enhancement by PSF (H3a) and PGF (H3b). Unlike leaders' OCBE in H2, leaders' WGGA is not a statistically significant predictor of members' WGGA. In contrast, for PSF and PGF, the presence of a main effect and the absence of a moderating effect mirror prior results (Table 3).

**TABLE 3:** Hierarchical linear modeling (HLM) results for H3a/b: Effect of team leaders' workgroup green advocacy (WGGA) × Person‐supervisor fit/Person‐group fit on team members' WGGA

DV: Members' WGGA	Est. (s.e.)	95% CI	*p* (2‐tail)		Est. (s.e.)	95% CI	*p* (2‐tail)
Intercept	−0.000 (0.076)	−0.144/0.143	0.999	Intercept	−0.007 (0.067)	−0.134/0.118	0.914
WGGA leader	0.103 (0.072)	−0.037/0.239	0.159	WGGA leader	0.083 (0.065)	−0.043/0.205	0.205
Member PSF (within teams)	0.185 (0.055)	0.077/0.292	<0.001 ***	Member PGF (within teams)	0.312 (0.052)	0.210/0.414	<0.001 ***
Member PSF (between teams)	0.194 (0.075)	0.052/0.336	0.012 *	Member PGF (between teams)	0.319 (0.068)	0.192/0.449	<0.001 ***
Leader female	−0.265 (0.190)	−0.623/0.096	0.170	Leader female	−0.224 (0.171)	−0.545/0.098	0.193
Member female	0.223 (0.153)	−0.068/0.523	0.147	Member female	0.217 (0.143)	−0.054/0.496	0.130
Age	−0.017 (0.007)	−0.031/‐0.004	0.014 *	Age	−0.012 (0.007)	−0.025/0.000	0.067
Educational level	−0.041 (0.084)	−0.201/0.129	0.630	Educational level	−0.055 (0.079)	−0.204/0.103	0.486
Number of team members	−0.005 (0.022)	−0.046/0.036	0.814	Number of team members	−0.013 (0.019)	−0.048/0.022	0.491
WGGA leader × PSF	0.002 (0.053)	−0.100/0.105	0.972	WGGA leader × PGF	−0.041 (0.047)	−0.133/0.052	0.390
Residual team‐level variance	0.063	Cond. *R* ^2^	0.222	Residual team‐level variance	0.029	Cond. *R* ^2^	0.294
Residual member‐level variance	0.797	Marg. *R* ^2^	0.160	Residual member‐level variance	0.721	Marg. *R* ^2^	0.266

Finally, in the fourth hypothesis, leaders' WGGA is the predictor of members' OCBE, with its effect assumed to be augmented by PSF (H4a) and PGF (H4b). The results are the same as for H3, notably with no main effect of leaders' WGGA and no moderating effect of PSF or PGF (see Table 4).

**TABLE 4:** Hierarchical linear modeling (HLM) results for H4a: Effect of team leaders' workgroup green advocacy (WGGA) × Person‐supervisor fit/Person‐group fit on team members' organizational citizenship behavior for the environment (OCBE)

DV: Members' OCBE	Est. (s.e.)	95% CI	*p* (2‐tail)		Est. (s.e.)	95% CI	*p* (2‐tail)
Intercept	0.012 (0.091)	−0.160/0.184	0.896	Intercept	−0.001 (0.081)	−0.153/0.152	0.995
WGGA leader	0.095 (0.081)	−0.060/0.248	0.246	WGGA leader	0.064 (0.073)	−0.076/0.203	0.386
Member PSF (within teams)	0.254 (0.047)	0.161/0.345	<0.001 ***	Member PGF (within teams)	0.311 (0.045)	0.222/0.399	<0.001 ***
Member PSF (between teams)	0.296 (0.085)	0.135/0.456	<0.001 ***	Member PGF (between teams)	0.415 (0.078)	0.267/0.563	<0.001 ***
Leader female	−0.105 (0.212)	−0.507/0.298	0.621	Leader female	−0.046 (0.192)	−0.410/0.317	0.810
Member female	0.067 (0.140)	−0.203/0.336	0.629	Member female	0.069 (0.133)	−0.190/0.323	0.604
Age	−0.014 (0.007)	−0.027/‐0.002	0.033 *	Age	−0.010 (0.007)	−0.022/0.002	0.113
Education level	−0.040 (0.077)	−0.185/0.117	0.601	Education level	−0.046 (0.073)	−0.185/0.101	0.528
Number of team members	−0.005 (0.027)	−0.056/0.046	0.852	Number of team members	−0.011 (0.023)	−0.055/0.034	0.651
WGGA leader × PSF	0.008 (0.045)	−0.079/0.096	0.854	WGGA leader × PGF	−0.029 (0.041)	−0.109/0.051	0.483
Residual team‐level variance	0.201	Cond. *R* ^2^	0.424	Residual team‐level variance	0.142	Cond. *R* ^2^	0.458
Residual member‐level variance	0.582	Marg. *R* ^2^	0.225	Residual member‐level variance	0.544	Marg. *R* ^2^	0.317

The results thus provide little support for our hypotheses. Neither PSF nor PGF moderates the influence of team leaders' WGGA and/or OCBE on members' WGGA and/or OCBE. In contrast, both PSF and PGF even more so are clearly associated with members' WGGA and OCBE. As for the main effects of leaders' WGGA and OCBE on members' WGGA and OCBE, a notable effect can merely be observed for leaders' OCBE (with statistically significant one‐tailed p values). However, including the country as an additional control variable weakens this effect of leaders' OCBE on members' WGGA and/or OCBE. In terms of demographics, our results suggest younger team members show a higher degree of WGGA and OCBE, with a considerable effect size as judged by the bivariate correlations (see Table 5) and a statistically significant association in several of the HLM models. In contrast, the effect of team size all but vanishes in the multivariate analyses. The effects of leader or member gender are inconclusive; their (contrary) effect is considered in the HLM results with WGGA as the criterion, but with too large standard errors to be statistically significant.

**TABLE 5:** Variable descriptives and correlations

		Mean	s.d.	1	2	3	4	5	6	7	8	9	10	11	12
1	Member OCBE	0.00	1.00	–											
2	Member WGGA	0.00	1.00	0.76	–										
3	Leader OCBE	0.00	1.00	0.34	0.28	–									
4	Leader WGGA	0.00	1.00	0.19	0.18	0.73	–								
5	PSF (within)	0.00	1.00	0.27	0.20	0.00	0.00	–							
6	PSF (between)	0.00	1.00	0.39	0.28	0.35	0.27	0.00	–						
7	PGF (within)	0.00	1.00	0.32	0.32	0.00	0.00	0.66	0.00	–					
8	PGF (between)	0.00	1.00	0.48	0.37	0.46	0.29	0.00	0.77	0.00	–				
9	Female leader	0.17	0.38	−0.04	−0.05	0.25	0.19	0.00	−0.06	0.00	−0.10	–			
10	Female member	0.22	0.42	0.04	0.10	0.16	0.04	0.06	0.02	0.03	0.01	0.40	–		
11	Member age	37.8	9.44	−0.32	−0.29	−0.38	−0.31	−0.08	−0.31	−0.08	−0.37	0.01	−0.10	–	
12	Member education	3.14	0.70	0.07	0.02	−0.02	−0.01	0.05	0.04	0.00	0.15	−0.17	−0.14	−0.11	–
13	n team members	6.26	3.92	0.20	0.14	0.20	0.33	0.00	0.47	0.00	0.46	−0.20	−0.15	−0.10	0.21

*Note*: n = 269, correlations ≥0.12 are statistically significant at the 5% level (two‐tailed).

An inspection of the fitted models with regard to the normality of residuals, homoscedasticity, and absence of influential outliers yields satisfactory results. Although some of the observed variables are highly correlated, these were not entered simultaneously as predictors into the HLM models. Therefore, collinearity was not an issue (VIF values are <2 in all instances). The pattern of results remains stable when taking the grand‐mean‐centered scores for PSF and PGF instead of including both within‐teams and between‐team effects. Likewise, using simple item sum scores instead of factor scores to calculate participants' values on the latent constructs does not change the results except in some models the main effect of leaders' WGGA and OCBE becomes marginally clearer. An additional analysis with a team‐level standard deviation of PSF as a predictor did not change the results, nor did PSF dispersion emerge as a notable predictor of members' WGGA/OCBE. Simultaneously entering both PGF and PSF and their interactions with WGGA and/or OCBE into the models results in only PGF remaining as a significant predictor and (still non‐significant) interaction effects with similar parameter estimates but opposite signs in all instances.

Despite the inclusion of the control variables mentioned earlier and the satisfactory results of the analyses regarding common method bias, our study admittedly remains a cross‐sectional survey with imperfect variable measurement and possibly omitted predictors and might thus suffer from endogeneity (Hill et al., 2021). In the absence of suitable instrumental variables, we examined this according to the procedure outlined by J.‐S. Kim and Frees (2007), specify the self‐reported predictor and moderator variables (leaders' WGGA/OCBE and PSF/PGF) as endogenous. Comparisons between the random effects estimator and the fixed effects estimator (and the generalized method of moment estimator) were never statistically significant, and our substantive results—notably the clear effect of PSF and PGF and the absence of our hypothesized interaction effects—remained stable across all analyses.

## 4 DISCUSSION

### 4.1 Theoretical contributions

This study makes two major contributions to the literature. First, recognizing the importance of employee support and participation for the success of transforming organizations toward sustainability, we examined the relationships between team leaders' and team members' pro‐environmental attitudes and behaviors. Albeit modest, the effects of team leaders' WGGA and OCBE on members' WGGA and OCBE (especially leaders' OCBE) indicate the influence of leaders' environmental commitment on employees' attitudes and behavior toward the environment, which is in line with LMX theory and previous research in the environmental management context (Cantor et al., 2015; Gkorezis, 2015). More importantly, the examination of the trickle‐down effects responds to a recent call for more research unpacking the multilevel process through which social conditions such as organizational policies and work‐team climate shape individual green behavior (Farrow et al., 2017; Norton et al., 2015). Our findings corroborate prior studies' reports of team leaders' role in promoting members' pro‐environmental behavior (A. Kim et al., 2017, Robertson & Barling, 2013).

From the human capital resource perspective, such positive green‐focused perception and behavior, as individual‐level KSAOs, can aggregate to create a group/unit‐level green‐focused human capital resource through an emergence enabling process (Ployhart & Moliterno, 2011). Our study demonstrates leaders' roles in promoting individual green‐focused KSAOs. Future research can go on to investigate how these individual KSAOs can combine in different ways (Ployhart et al., 2014) to form collective emergence‐enabling cognitive and behavioral states that form, in conjunction with the task environment, a green human capital resource for a firm's green performance and competitive advantage (Eckardt et al., 2021).

An additional analysis of our data based on the bivariate correlations (see Table 5) shows that leaders' OCBE is more strongly associated with members' WGGA and OCBE than leaders' WGGA. A test for dependent correlations corroborates this association. Apparently, leaders' OCBE, functioning as a type of instrumental support in the social exchange process, shows a stronger influence on followers than leaders' WGGA which serves as a type of emotional support. These results are consistent with Paillé et al. (2020), who found that instrumental support predicted employees' OCBE more consistently than emotional support did.

Second, corresponding to a call to consider different dimensions of PEF to identify their influence on behavioral and attitudinal constructs (Jansen & Kristof‐Brown, 2006), we simultaneously investigated PGF and PSF relating to the transfer of green engagement in a team context. Contrary to our assumptions, we observed no moderation effects of members' fit with their supervisors or other members. Even for the observed effect that deviates farthest from zero (leaders' WGGA and PGF on members' WGGA), using the *R* ^2^ values for the model with and without the interaction results in an *f* ^2^ of 0.001 (an *f* ^2^ of 0.02) is a small effect, cf. Cohen (2013). A potential explanation for the observed absence of any notable moderator effects of PGF or PSF could be because of conflicting mechanisms in the PEF framework (Seong & Choi, 2021). As part of the PEF, this study assessed the value fit of employees (as opposed to ability fit) through the dimensions of PGF and PSF (see the Measures section). On the one hand, social identity notions might foster a reinforcing effect of such supplementary fit on the relationship between team leaders' and members' WGGA or OCBE. On the other hand, normative pressure toward conformity stemming from the same value fit might dampen the leaders' influence on members' green initiatives such as WGGA or OCBE for fear of “unduly outperforming” one's peers and/or supervisors.

Yet another explanation is that leaders' OCBE and WGGA, when affecting members' OCBE and WGGA, already involve social exchanges featured with supervisory support and mutual obligations. As such LMX processes unfold, they enhance members' quality relationships with their leaders, resulting in perceived fit with their immediate social environments (i.e., fit with leaders and peers). Therefore, the main effects of leaders' OCBE and WGGA reported in the regression analyses may already absorb some effects of perceived fit. Moreover, because there is still a lack of multilevel research regarding the interactive effects of PEF dimensions (Ostroff & Schulte, 2007), future research should especially consider the impact of value fits and the pressure of conformity to clarify the interaction of the various PEF dimensions with leadership variables in predicting employees' environmental performance.

Surprisingly, our findings confirm that PSF and PGF can be regarded as antecedents for WGGA and OCBE in an organization. Both PSF and PGF emerge as significant predictors of members' WGGA and OCBE, which accords with findings on the importance of connectedness for employees to show positive environmental attitudes and behavior (Cojuharenco et al., 2016). Additionally, our results are in line with previous research on the effect of value congruence on attitudinal outcomes (Cable & Edwards, 2004) and on the influence of PEF dimensions on voluntary behaviors (Farzaneh et al., 2014). From a social exchange perspective, it is imaginable that the perceived value congruence between a leader and members as indicated by PSF is in itself already construed by members as an affective contribution to the dyadic relationship on the part of the leader. In turn, this construal causes them to reciprocate by increasing their behaviors that exceed formal job requirements (Maslyn & Uhl‐Bien, 2001), such as OCBE. Nevertheless, an explorative comparison of the bivariate correlations indicates that PGF is a stronger predictor than PSF for both team members' and team leaders' voluntary environmental initiatives. A possible explanation could be that if individuals perceive their values, including those concerning the environment, to be in accordance with their group members' values, they may feel more confident and reassured in openly expressing related behaviors, attitudes, and advocacy. In light of the strong connection between PGF and group cohesion, job satisfaction, and organizational commitment (Kristof‐Brown et al., 2005), it could be investigated whether and how these variables shape the positive influence of the fit with one's group on individual outcomes related to the environment.

### 4.2 Practical implications

From our findings, we derived four practical implications for companies that seek to establish or advance pro‐environmental strategies. First, our finding of a perceptible effect of leaders' OCBE on members' OCBE and WGGA suggests managers' positive environmental beliefs should be fostered through HR practices in order to achieve a lasting change in an organization toward sustainability (Ren, Jiang, & Tang, 2022). HR managers, who want to foster pro‐environmental behaviors and attitudes in their organizations, can benefit from our findings by focusing on supervisors' and team members' supportive attitudes. Employees' WGGA positively correlates with employees' OCBE and drives environmental performance, advancing organizations toward their sustainability goals. In addition, members' WGGA promotes holistic pro‐environmental activities inside and outside of an organization, such as supply chains and local communities, thereby advancing environmental activities beyond intra‐company strategy, which in turn leads to better environmental performance (Edwards & Kudret, 2017).

Second, our data show that PSF and PGF can be regarded as antecedents for WGGA and OCBE in an organization. HRM measures should target strengthening the PGF in teams. Such strengthening has several beneficial outcomes, such as job satisfaction, organizational commitment, and socially responsible behavior (Cojuharenco et al., 2016; Kristof‐Brown et al., 2005). Therefore, practical interventions to improve employees' green behavior and the sustainability performance of companies should focus first on leaders because they act as distributors of environmental sustainability behavior within their teams (Wang et al., 2020). Organizations may promote an inclusive work environment in which people feel they and their supervisors are fitting into the group as indicated by PSF and PGF (de Cooman et al., 2016). Recognizing diversity as a degree of divergence and managing inclusiveness through managerial commitment, targeted communication, and enterprise resource groups have a significant impact on inclusion and PGF (Burns & Ulrich, 2016). By highlighting the influence of PGF as a strong predictor of pro‐environmental behaviors and attitudes on the part of leaders and team members, this study also implies that HRM should focus not only on the managerial level but also include a multilevel perspective by considering nonmanagerial employees as well (Ren & Jackson, 2020). By taking into account the interpersonal aspects of a pro‐environmental stance across organizational levels, a holistic impact on the transformation of organizations toward sustainability can be achieved.

Third, the lack of a moderating effect of PSF or PGF on the relationships between team leaders' OCBE/WGGA and team members' OCBE/WGGA implies practice that activities to encourage OCBE and WGGA through the motivation of team leaders can be performed independently from activities to encourage OCBE and WGGA via programs on PGF/PSF. This has practical implications because inclusion measures to drive PGF and PSF are often implemented by different organizational entities (Diversity and Inclusion) than those entities charged with driving leaders' support of OCBE and WGGA (Sustainability).

Fourth, we found that, although correlations vary, they are still valid across all examined regions and countries. These correlations across countries can help practitioners in MNCs to balance global and local environmental strategies in their decision making to advance their environmental goals. Companies that define a global strategy to promote PSF, PGF, OCBE, and WGGA can expect an overall positive impact on environmental activity and goal advancement.

### 4.3 Limitations and directions for future research

Although our findings are clear both in terms of main effects and the absence of moderating effects, our study has several limitations and raises questions for future research. Despite using extant construct operationalization with good internal consistency values, the psychometric properties of the scales are satisfactory in most but not all aspects of the present sample, such as measurement invariance between countries or uncorrelated residuals. Also, despite being conceptually distinct and showing adequate discriminant validity, OCBE and WGGA were highly correlated on both the leaders' and members' levels.

Because we used a cross‐sectional survey design, proposed relationships can only be assumed and drawn from theory and literature. Also, despite the satisfactory results of the analyses regarding endogeneity, it cannot be categorically ruled out. Future research should include longitudinal designs to further validate the effects of attitudes and behavior of supervisors on the environmental actions and performance of teams. Although the general link of these variables seems widely accepted in the research community, the specific circumstances under which the leader‐member interaction is promoted or hindered should be examined in‐depth in future research. To fully understand the relationships between employees and leaders, when it comes to the impact of employees' environmental attitudes and behavior on their leaders, it will be necessary not only to follow the top‐down perspective but also to account for the impact of employees on their leaders in a bottom‐up approach.

We collected data via surveys and relied on self‐reported information (although from two different sources for leaders versus members' OCBE and WGGA, thus reducing the risk of common method/single source bias on a design level). Although this seems less problematic for attitudes (Gawronski et al., 2006), we realize the potential discrepancies in self‐reported OCBE versus actual OCBE (Kormos & Gifford, 2014). Additional work is needed to validate the environmental performance of teams and subsidiaries based on self‐reported team members' OCBE. Another aspect of the survey that turned out to be a possible limitation was the lack of moderating effects from the PEF dimensions, that is, PGF and PSF. Because PGF and PSF are the value dimensions of the PEF framework (Seong & Choi, 2021), social norms and the expectations of peers and supervisors might influence the effect of PGF and PSF on team members. Further research should consider these possible effects when planning a survey and also examine the interactions of the PEF dimensions and the contextual conditions (Oh et al., 2014).

The special setting of our study—one MNC across different subsidiaries and countries—allowed us to broaden the geographical dispersion of OCBE research (Yuriev et al., 2018). Although we focused on one MNC to ensure a consistent organizational environment across teams, further research can evaluate different types of organizations such as small‐ and medium‐size enterprises or public service entities (Wang et al., 2020).

## 5 CONCLUSION

In this study, we investigated how the interaction of teams and leaders in different institutional contexts plays a role in changing organizations toward sustainability. In addition, we analyzed how pro‐environmental behaviors and attitudes within a team may be affected by a perceived fit with the team or team leader. To do so, we examined the moderating role of PSF and PGF on the influence of team leaders' OCBE and WGGA on members' OCBE and WGGA in an MNC. Instead of a moderating effect, our results show a predictive role for leaders' OCBE and WGGA—as well as employees' PSF and PGF—on employees' perception and behavior toward the environment. Furthermore, our significant and consistent findings allow concluding that HR policies should aim to foster PGF with team members and support leaders' OCBE. As MNCs deploy global environmental strategies and implementation to pursue an organization's transformation toward sustainability, our findings point to the importance for organizational leaders to become aware of the supporting effects in order to tackle this grand challenge. Our study contributes to HRM practice by confirming, across regions, the effectiveness of environmental programs' focus on leaders and teams.

## Data Availability

The data that support the findings of this study are available on request from the corresponding author. The data are not publicly available due to privacy or ethical restrictions.

## Endnote

^1^
First item pair: I encourage my colleagues to adopt more environmentally conscious behavior/I encourage my colleagues to express their ideas and opinions on environmental issues. Second item pair: I actively participate in environmental events organized in and/or by my company/I volunteer for projects, endeavors or events that address environmental issues in my organization.
